# Schmidt on Yellow Fever

**Published:** 1881-05

**Authors:** S. V. Clevenger


CHICAGO MEDICAL

JOURNAL & EXAMINER.

VOL. XLII.—MAY, 1881.—NO. 5.

### Original Communications.

ARTICLE I.

SCHMIDT ON YELLOW FEVER. Read before the Chicago Biological Society, April 6, 1881. By S. V. CLEVENGER, M.D.

The medical world is not aware that an era has been reached in general pathology, but it is indeed true. The justly distinguished scientist and physician, H. D. Schmidt, Pathologist of Charity Hospital, New Orleans, has gathered together the results of a labor extending through a number of years, in a volume entitled, "The Pathology and Treatment of Yellow Fever."

To glean an idea of the bearing this forthcoming work will have upon science and medicine, and to convince you of its value as a contribution to pathology in general, I must be allowed to present a little biography of its author, indicative of the route by which he approached his subject and his acquisition of the necessary scientific, literary and logical training, prefacing the little history by the statement that it required considerable effort to gather together the facts, and only by a threat of publishing the incomplete and less accurate details, could Dr. Schmidt be induced to revise the biography and furnish a list of his works.

Leaving aside his unguided studies of natural history during his boyhood, his systematic education began in 1853, with anatomy, chemistry and physiology, to which subsequently was added botany and comparative anatomy. His inherent inclination to the study of Nature in general led him to medicine, so he was in no hurry to obtain a diploma, and in consequence left the more practical branches of our profession untouched until 1856, and two years later he graduated in medicine. His familiarity from boyhood with the three great living languages, facilitated his acquisition of a classical education. Anatomy being his favorite study, he pursued it in all its various departments, special and microscopical, experimented in minute injections until he had become a master, made all sorts of preparations, and thus could not fail to become an anatomist in the fullest sense of the word.

Under the auspices of his good friend, Prof. Joseph Leidy, of Philadelphia, he constructed many large anatomical models, which may now be seen in the old University of Pennsylvania. These models won for him a wide reputation. Aided by Prof. Samuel Jackson, of that school, he entered upon microscopical anatomy in the spring of 1857, Prof. Leidy placing his "Natchet" microscope at his disposal. In Schmidt's paper on the hepatic lobule will be found a description of his microtome, invented for the original purpose of making sections of spinal marrow, but aiding him afterwards in discovering the biliary capillaries as the commencement of the hepatic duct. (*American Journal of Medical Science*, January, 1859.)

Queckett's section cutter being only for wood, it is probable that Schmidt's microtome was the first ever constructed for making sections of soft tissues. In 1857 he made a considerable number of experiments and observations for Prof. Jackson, on living animals—dogs, cats and hogs—concerning the digestion of the muscular fibers in the alimentary canal. After graduation, Schmidt was appointed Leidy's assistant and prosector, the same position which the latter once held under Prof. Horner. At the same time Schmidt acted as assistant demonstrator under Dr. Wm. Hunt. During 1858 and 1859 he was engaged in his researches into the histology of the liver, for which work he invented the most useful and perfect injecting apparatus that ever has been seen.

In February, 1860, Schmidt left Philadelphia, being called to the Medical college of Alabama, by his friend, the celebrated Dr. J. C. Nott, of Mobile. Soon after he was induced to accept the position of prosector and demonstrator of anatomy in the New Orleans School of Medicine, at $1,500 per year (a large sum at that time), and he thus became a permanent resident of New Orleans. The civil war broke out, and a blank of five years was made in Schmidt's scientific career. Circumstances (above which the best and strongest are unable to swim, say what we may) found him in the Confederate army in the capacity of surgeon, without books or instruments, cut off from the scientific world. He spent his time in the discharge of his professional duties in hospital and camp, or in the neighboring forests in the study of plants and animals, often astonishing his surgeon-colleagues by his dissections of spiders or bony ears of mammals and amphibia by means of his pen-knife.

Upon returning to New Orleans, he was appointed Pathologist of the Charity Hospital, and besides this, he temporarily occupied the chair of Physiology in the New Orleans Medical School for Dr. A. C. Holt, who was prevented from lecturing by sickness. In the spring of 1866, Schmidt was attacked by rheumatism, which compelled him to resign his hospital position. In May, 1867, he regained health sufficiently to recommence general practice, and resume his favorite scientific studies, which he continued till 1874, when his old enemy, rheumatism, overtook him again, laying him up for seven long months and disabling him forever for the active duties of his profession. Dr. Schmidt is so very much disabled that it is positively painful to watch him endeavoring to make his old time delicate dissections and microscopic manipulations. Nevertheless he manages successfully to make as good sections and slides as any microscopist, and his drawings are faultless, both as to faithfulness to nature and artistic finish. The specimens I here exhibit were made by him, and they have elicited the admiration of our Illinois Microscopical Society, Profs. J. S. Jewell, Lester Curtis, Wm. A. Hammond, E. C. Seguin, E. C. Spitzka and others.

During his prostration, Schmidt wrote his American Neurological Society paper, on the "Structure of the Nervous Tissues and their Mode of Action." In October, 1877, just as his means were exhausted, by application of the most prominent physicians of New Orleans, he was re-appointed Pathologist of Charity Hospital, and it was in this capacity that he made his more recent investigations into the pathology of yellow fever, bringing to bear upon his researches the results of observations made during previous epidemics and the great skill and learning acquired through a life-long, earnest search for truth.

Schmidt's principal papers are:

1. "On the Minute Structure of the Hepatic Lobule, Particularly with Reference to the Relationship between the Hepatic Cells and the Canals which carry off the Secretion of the latter." (Illustrated with Thirty-three Figures.) Published in the *American Journal of Medical Sciences*, January, 1859. (This paper contains his discovery of the biliary capillaries.)

2. "Researches into the Pathology and Cause of the Present Epidemic, Ordinarily Called 'Yellow Fever.'" Published in the *Southern Journal of Medical Sciences*, November, 1867. (Written during the epidemic).

3. "Microscopical Anatomy of the Human Liver. (Illustrated with Three Plates.) Published in the New Orleans *Journal of Medicine*, October, 1869, January and April, 1870.

4. "On the Origin and Development of the Colored Blood Corpuscles in Man." (Illustrated by Ten Figures.) Read before the Royal Microscopical Society of London, and published in its former organ, the *Monthly Microscopical Journal*, 1874.

5. "On the Construction of the Dark or Double-bordered Nerve Fiber. (Illustrated by Twenty-two Figures.) Published by the Royal Microscopical Society of London, *Monthly Microscopical Journal*, May, 1874.

6. "Synopsis of the Principal Facts, Elicited from a Series of Microscopical Researches upon the Nervous Tissues." Read before the Royal Microscopical Society of London, and published in the *Monthly Microscopical Journal*, July, 1874.

7. "On the Development of the Smaller Blood Vessels in the Human Embryo." (Illustrated by Nineteen Figures.) Read before the Royal Microscopical Society of London, and published in the *Monthly Microscopical Journal*, January, 1875.

8. "On the Structure of the Nervous Tissues and their Mode of Action." Published in the Transactions of the American Neurological Society, Vol. 1, 1875.

9. "The Development of the Nervous Tissues of the Human Embryo." (Illustrated by Twenty-six Figures.) Published in the *Journal of Nervous and Mental Disease*, July, 1877.

10. "The Structure of the Colored Blood Corpuscles of *Amphiuma Tridactylum*, the Frog and Man." (Illustrated by Fifty-eight Figures.) Taken as read before the Royal Microscopical Society of London, and published in the Journal of the Royal Microscopical Society, May and July, 1878.

11. "Case of Repeated Attacks of Apoplexy, with Aphasia." (Illustrated by Five Figures.) Published in the *Journal of Nervous and Mental Disease*, July, 1878.

12. "On the Structure and Function of the Ganglionic Bodies of the Cerebro-Spinal Axis." (Illustrated by Fifteen Figures.) Published in the *Journal of Nervous and Mental Disease*, January, 1879.

13. "On the Pathology of Yellow Fever." Published in the New York *Medical Journal*, February, 1879.

14. "On the Nature of the Poison of Yellow Fever and its Prevention." Published in the New York *Medical Journal*, May, 1879.

Dr. Schmidt has a host of warm, personal friends in the profession in all parts of the world. His researches are standard topics in our better class of text-books.

It is notorious that no one can succeed as a scientific man, and at the same time earn even a respectable living, without assistance. It is in obedience to the law of labor division. How is it possible for a nerve cell to exist if the blood corpuscle did not carry it oxygen, and the tissues from which it emanated afford it support? Dr. Schmidt has been so engrossed in his scientific labor that to-day he finds himself fifty-six years of age, prostrated in health, with a family to support, and very little to cheer his declining years in the way of a pecuniary outcome for work that not one man in ten million is capable of doing, and few indeed have done so earnestly and honestly, and with such purity of motive. In a few hundred years the world will appreciate these delvers after truth, who are content with truth only and nothing else, and who in their detestation of humbuggery, avoid the conspicuousness which leads to affluence.

The merits of the work on Yellow Fever, which I here present, you may judge for yourselves.

It is through its sale by subscription and wholly upon its merits as a scientific work, and as a genuine contribution to knowledge, that some recompense is expected for this life of toil.

---

At a meeting of the Chicago Biological Society, April 6, 1881, after the presentation of the foregoing paper, accompanied with lithographs and microscopic slides prepared by Dr. Schmidt, extracts from his manuscript work were read, and the following resolution passed:

"*Resolved*, That the Society cordially endorse the work of Dr. Schmidt, and recommend the publication of the same by subscription under its auspices; and that its treasurer be made the treasurer of the necessary subscription fund, and be held responsible to Dr. Schmidt for all subscriptions received by the former."

It was also "*Resolved*, That Dr. Clevenger be requested to publish his paper, as read during the evening, in the Chicago Medical Journal and Examiner, as preliminary to the appearance of Dr. Schmidt's work.

In accordance with the above resolution, the work of Dr. Schmidt will appear in monthly serial parts in the Chicago Journal and Examiner until completed.

*The subscription price will be* $3.00, *which may be sent either directly to* Dr. H. D. Schmidt, 263½ *Canal St., New Orleans, La., or to* Roswell Park, m.d.,

*Sec'y and Treas. Chicago Biological Society,*
1558 Wabash Ave., Chicago.

